# Regenerated Microvascular Networks in Ischemic Skeletal Muscle

**DOI:** 10.3389/fphys.2021.662073

**Published:** 2021-06-11

**Authors:** Hao Yin, John-Michael Arpino, Jason J. Lee, J. Geoffrey Pickering

**Affiliations:** ^1^Robarts Research Institute, Western University, London, ON, Canada; ^2^Department of Medicine, Western University, London, ON, Canada; ^3^Department of Medical Biophysics, Western University, London, ON, Canada; ^4^Department of Biochemistry, Western University, London, ON, Canada

**Keywords:** skeletal muscle, peripheral artery disease, angiogenesis, intravital microscopy, smooth muscle cell

## Abstract

Skeletal muscle is the largest organ in humans. The viability and performance of this metabolically demanding organ are exquisitely dependent on the integrity of its microcirculation. The architectural and functional attributes of the skeletal muscle microvasculature are acquired during embryonic and early postnatal development. However, peripheral vascular disease in the adult can damage the distal microvasculature, together with damaging the skeletal myofibers. Importantly, adult skeletal muscle has the capacity to regenerate. Understanding the extent to which the microvascular network also reforms, and acquires structural and functional competence, will thus be critical to regenerative medicine efforts for those with peripheral artery disease (PAD). Herein, we discuss recent advances in studying the regenerating microvasculature in the mouse hindlimb following severe ischemic injury. We highlight new insights arising from real-time imaging of the microcirculation. This includes identifying otherwise hidden flaws in both network microarchitecture and function, deficiencies that could underlie the progressive nature of PAD and its refractoriness to therapy. Recognizing and overcoming these vulnerabilities in regenerative angiogenesis will be important for advancing treatment options for PAD.

## Introduction

Skeletal muscle is the largest organ in humans, constituting roughly 40% of the body mass. The role of skeletal muscle in powering voluntary movement demands a system that tightly couples muscle metabolism with blood flow. Much of this control system is local, wherein myofiber oxygen requirements are met through tuning microvascular flow dynamics (Ellis et al., 2005; Segal, 2005). The attributes of this flow control system include a microvascular architecture that is optimally organized among the skeletal myofibers, and a set of physiologic systems that regulate arteriolar tone, red blood cell transit in capillaries, and oxygen delivery.

However, the health of skeletal muscle, and the associated microvasculature, can become profoundly compromised as we age. An extreme, common, and understudied example of this is peripheral artery disease (PAD). The hallmark of PAD is the atherosclerotic narrowing of the large and medium-sized arteries of the lower limbs. However, the downstream consequences include ischemia and death of skeletal myofibers (Regensteiner et al., 1993; White et al., 2016; Mcdermott et al., 2020) and a compromised microcirculation (Hillier et al., 1999; Coats and Hillier, 2000; Robbins et al., 2011; Baum et al., 2016; Chevalier et al., 2020). When widespread and severe, the damage from PAD is unremitting and amputation is required.

As efforts are made to avoid the dire outcomes of PAD, an important consideration is a long-recognized fact that skeletal muscle has the capacity to regenerate (Charge and Rudnicki, 2004). Similarly, the vasculature has the capacity to reconfigure as part of the drive to ensure muscle viability and function following ischemic insult. This includes the recruitment of endogenous collateral vessels as an upstream response, and regenerative angiogenesis as a downstream response (Hudlicka et al., 1992; Wagner, 2001; Olfert et al., 2016). Collateral vessels are artery-to-artery or arteriole-to-arteriole connections that are engaged when an upstream limb is obstructed, yielding a pressure gradient across the collateral network (Faber et al., 2014). Regenerative angiogenesis on the other hand entails the formation of new microvessels, primarily capillaries, to supply the regenerating tissue.

In this perspective, we review how the skeletal muscle microvasculature is affected by, and responds to, ischemic injury. We summarize the elements of the microvasculature in normal skeletal muscle, and we highlight recent advances in understanding the architecture and function of a regenerated distal microvasculature in skeletal muscle subjected to ischemic injury. We discuss how emerging data can reduce the knowledge gaps pertaining to the regenerated microcirculation in ischemic muscle, with relevance to PAD.

## Microvascular Architecture in Skeletal Muscle

The microcirculation consists of blood vessels below ~200 μm diameter (Levy et al., 2001) and is responsible for locally delivering oxygen (O_2_), nutrients, and circulating signals to tissues, while also removing tissue metabolites (Pries et al., 1995; Segal, 2005). The cell components of the microvessel wall depend on the site within the hierarchy – i.e., arteries, arterioles, capillaries, venules, and veins – but consist of a monolayer of endothelial cells (ECs) surrounded by one or more layers of mural cells. ECs are the primary sensor for the biochemical and biomechanical signals arising from the flowing blood (Cunningham and Gotlieb, 2005). The mural cells, smooth muscle cells (SMCs) around arterioles and pericytes around capillaries, collectively serve to regulate vascular tone, flow control, capillary stability, and barrier function (Segal, 2005; Holm et al., 2018; Gonzales et al., 2020).

Feed arteries penetrate the skeletal muscle epimysium and branch into an arcade-like network of interconnected arterioles within the perimysial space. This network gives rise to transverse arterioles which enter the endomysium and divide into higher-order arterioles, and ultimately the terminal arterioles. Terminal arterioles, in turn, branch into a meshwork of capillaries (Holley and Fahim, 1983; Engelson et al., 1986; Dodd and Johnson, 1991). The precise topology of the capillary meshwork is adapted to the anisotropic arrangement of skeletal muscle (Emerson and Segal, 1997). Capillaries run parallel to the long axis of skeletal myofibers and course for approximately 0.5–1 mm in length between myofibers before draining into myofibers. Interestingly, the terminal arteriole gives rise to two capillary beds coursing in opposite directions. The microvascular unit thus entails the terminal arteriole and the capillaries it supplies in both directions. Post-capillary venules are positioned between two terminal arterioles and collect blood from the corresponding two capillary beds (Delashaw and Duling, 1988; Ellis et al., 2005; Olfert et al., 2016). Also important are frequent anastomoses between parallel capillary segments (Emerson and Segal, 1997). This interconnected capillary network is central to the intrinsic heterogeneity of RBC perfusion that exists among different capillaries fed by the same arteriole, a fundamental feature that enables regulated flow distribution to the myofibers (Duling and Damon, 1987; Ellis et al., 1994).

## Regulation of Microvascular Flow in Skeletal Muscle

Skeletal muscle has an astonishing capacity to increase its blood flow, which can augment over 100-fold (Laughlin and Armstrong, 1982; Andersen and Saltin, 1985; Thomas and Segal, 2004; Saltin, 2007). Increased blood flow during muscle activity is orchestrated by adjustments in heart rate and cardiac contractility, with baroreflex-mediated vasoconstriction in peripheral organs redistributing blood flow to contracting muscles (Stubenitsky et al., 1998). Within the skeletal muscle microvasculature under resting conditions, resistance vessels maintain spontaneous vasomotor tone (Duling and Damon, 1987; Segal et al., 1989; Segal, 2005). Increased flow in skeletal muscles is sensed by arterial ECs which stimulate the endothelial release of vasodilatory agents like nitric oxide (NO) and prostaglandins (Clifford, 2011). These agents instruct arterial/arteriolar SMCs to relax, decreasing resistance and augmenting flow (Welsh and Segal, 1998). Vasomotor tone controlled at the terminal arteriole is important in distributing RBCs into specific capillary beds, in accordance with the regional demands of myofibers (Duling and Damon, 1987; Koller et al., 1987; Pries et al., 1989; Frame and Sarelius, 1993; Ellis et al., 1994; Kusters and Barrett, 2016).

One important basis for matching regional myofiber demands with RBC delivery is the sensing of tissue hypoxia. Hypoxia sensing can occur at the capillary level (Parthasarathi and Lipowsky, 1999; Jia et al., 2011), and in some settings by SMCs (Franco-Obregon et al., 1995), and initiate a vasodilation response that increases perfusion. The specific signaling elements mediating oxygen-based reactivity may vary based on region (Jackson and Duling, 1983; Jackson, 1993). However, an evolving paradigm for hypoxia-induced vasodilation entails deoxygenated RBCs in capillaries releasing ATP to activate purinergic receptors on closely apposed ECs. The resultant signals are conducted upstream, through gap junctions, as vasodilatory signals for arterioles (Ellsworth et al., 1995; Ellsworth, 2004; Ellis et al., 2012; Leybaert and Sanderson, 2012). A complementary paradigm posits that capillaries are both positioned and equipped to receive signals originating from contracting skeletal muscle fibers. Again, the capillary endothelial cells then transmit these signals upstream to the arteriolar vasculature to augment flow (Murrant et al., 2017). In both scenarios, the phenomenon of conducted vasodilation is central. Hyperpolarization signals travel up the microvascular tree, mainly *via* endothelial gap junctions, and are conveyed to arteriolar SMCs through myoendothelial gap junctions. The resultant vasorelaxation augments flow to precisely those muscle territories in demand (Bagher and Segal, 2011; Murrant et al., 2017).

## The Microvasculature in Ischemic Skeletal Muscle: A Knowledge Gap for PAD Management

Disease-associated defects in the skeletal muscle microcirculation are well recognized. For example, skeletal muscle capillary rarefaction can occur in chronic kidney disease and heart failure (Hendrickse and Degens, 2019; Querfeld et al., 2020), and microvascular structure and function in skeletal muscle can be impaired in diabetes and sepsis (Bateman et al., 2008; Horton and Barrett, 2021). As noted, another important disease context for a compromised microcirculation is PAD, where the upstream large and medium-sized arteries themselves are diseased. PAD is a major health concern. It is the third leading cause of cardiovascular mortality in developed countries and affects up to 20% of individuals over 70 years of age (Gerhard-Herman et al., 2017; Aboyans et al., 2018). A critical and severe manifestation of PAD is chronic limb-threatening ischemia (CLTI). This is a condition characterized by intractable pain, non-healing ulcers, and tissue necrosis. There is no effective medical treatment for CLTI, and endovascular and surgical revascularization strategies are employed (Gerhard-Herman et al., 2017; Aboyans et al., 2018). However, up to a third of CLTI patients who have undergone an intervention still require major amputation within 3 years (Almasri et al., 2019).

Different components of the microvasculature may be impacted in PAD. Native, small arteries harvested from patients with CLTI have been reported to display wall thinning, as well as impaired vasomotor responses to acetylcholine and nitroprusside (Hillier et al., 1999). Impaired vasomotor responses of feed arteries have been identified in the mouse following femoral artery resection (Cardinal et al., 2011). Compromised vascular reactivity in PAD subjects has also been suggested by the finding of reduced plasma nitrite responses to exercise (Allen et al., 2009). Also important are variations in the abundance, remodeling, and function of collateral vessels, key determinants of perfusion in PAD (Bailey et al., 2008; Mac Gabhann and Peirce, 2010; Ziegler et al., 2010).

Data on the status of capillaries in PAD are limited and restricted to histologic assessment. Capillary basement membrane thickening has been reported in subjects with moderate PAD, and increased collagen and pericyte content around capillary endothelial cells has been noted in subjects with severe PAD (Roos et al., 2016; Mietus et al., 2020). Capillary lumen size itself has been reported to be normal in patients with intermittent claudication (Baum et al., 2016, 2020). Some studies have reported a modest decrease in capillary density in PAD skeletal muscle (Clyne et al., 1985; Robbins et al., 2011). However, several other studies have identified increased capillary counts in PAD skeletal muscle (Hammarsten et al., 1980; Mcguigan et al., 2001; Tsui et al., 2002; Ho et al., 2006a,b; Chevalier et al., 2020).

This latter finding is important because it indicates an angiogenic response in patients with PAD. However, beyond capillary counting, there is little known about the regenerated microvasculature that forms after ischemic insult, with limited data on network architecture and function. This is also the case for animal models, including mouse models of ischemic skeletal muscle where robust angiogenesis is well documented (Limbourg et al., 2009). The paucity of functional data is particularly noteworthy recognizing that stimulating angiogenesis as a therapeutic tool has garnered considerable interest, but one that has not translated into benefit for patients with PAD (Iyer and Annex, 2017). A better understanding of angiogenesis in ischemic muscle is thus a priority.

## Angiogenesis in Ischemic Skeletal Muscle

In the embryo, vascular morphogenesis proceeds in two primary steps – vasculogenesis and angiogenesis. Vasculogenesis entails the *de novo* production of an EC-lined primitive vascular plexus from progenitor cells (Marcelo et al., 2013). This process proceeds early in embryogenesis, with angioblasts differentiating from mesoderm to form the primordial blood vessels (Patel-Hett and D'Amore, 2011). Angiogenesis is the formation of new microvessels from preexisting ones. It follows vasculogenesis in the embryo and underlies the formation of most of the blood vessels (Patel-Hett and D'Amore, 2011). Some features of vasculogenesis have been identified in ischemic adult muscle (Tamaki et al., 2005; Leroyer et al., 2009; Patel et al., 2017). However, angiogenesis is a well-established process in adult tissues that are remodeling, repairing, and regenerating (Herbert and Stainier, 2011), including ischemic skeletal muscle (Olfert et al., 2016; Iyer and Annex, 2017).

Mouse models of ischemia, wherein flow down the femoral artery is halted, have been extensively used to investigate angiogenesis in adult skeletal muscle (Couffinhal et al., 1998; Limbourg et al., 2009). The ischemic injury in these models is acute and typically strong, particularly if the femoral artery is excised or multi-ligated (Limbourg et al., 2009). As such, the effects of more indolent, chronic hypoperfusion on skeletal muscle microvessels, as may exist in patients with PAD, are not captured. Instead, the flow-cessation models provide a valuable opportunity to interrogate the angiogenic and regenerative capacity of the ischemic lower limb, and the effect of interventions that might enhance or suppress the regenerative cascades.

Angiogenesis in skeletal muscle is commonly identified histologically and quantified based on the density of capillaries or the capillary-to-myofiber ratio. These endpoints have been extensively studied in response to exercise (Waters et al., 2004; Egginton, 2009) but also employed in ischemic models (Olfert et al., 2016; Said et al., 2019; Lee et al., 2020). A caveat to these particular assessments is that they do not account for differences in muscle fiber size or metabolic subtype. Relating capillary counts to the area or perimeter of the specific myofiber with which they are in contact may more effectively convey capillary supply (Latroche et al., 2015). Nevertheless, as a global histological parameter, capillary density is useful for evaluating if angiogenesis has occurred following an ischemic insult and if an intervention can modify the response.

Another critical consideration when histologically quantifying capillary content in the hindlimb is the spatial heterogeneity of ischemic injury. Skeletal muscle occupies a large territory of the mouse hindlimb, with over 20 different muscles (Kochi et al., 2013; Charles et al., 2016) and with different myofiber subtypes (Cherwek et al., 2000; Zaccagnini et al., 2015). As well, the extent and location of ischemic injury can vary based on variations in technique and inter-mouse differences in collateral responses (Helisch et al., 2006; Limbourg et al., 2009). Given these issues, the potential for regional heterogeneity in an angiogenic response is considerable. To investigate this spatial heterogeneity, we recently generated an atlas of myogenesis and angiogenesis in the C57Bl/6 mouse hindlimb following excision of the femoral artery (Lee et al., 2020). This analysis revealed that angiogenesis, as quantified from capillary densities in muscles across the entire hindlimb at different planes, was variable and surprisingly patchy. Neovascularization was most reliably found in the distal anterior hindlimb muscles, including the tibialis anterior and external digitorum longus muscles. Also important is that angiogenesis was identified exclusively in regions of skeletal muscle that had undergone infarction followed by regeneration, as denoted by central myofiber nuclei. Non-regenerated muscle zones, including border zones, displayed no angiogenesis (Lee et al., 2020).

Surprisingly, a systematic review of 509 peer-reviewed manuscripts investigating angiogenesis following femoral artery excision in C57BL/6 mice revealed that the approach to histological quantitation of angiogenesis was often discordant with the above mapping data. For example, in only 7% of the studies reviewed was angiogenesis assessed in the distal anterior hindlimb, a high-likelihood region. This does not exclude the possibility that the muscles studied had angiogenesis but it raises uncertainty. Moreover, among the 509 manuscripts reviewed, in only 15% of studies was there a consistent depiction of central nuclei (i.e., regenerated skeletal muscle) in representative post-injury hindlimb images (Lee et al., 2020).

Thus, there is room for increasing the reliability of assessing post-ischemia angiogenesis by focusing on those territories where myofibers have central nuclei. That said, even with a rigorous methodological framework, it must be recognized that the histological evaluation of angiogenesis provides limited opportunity for ascertaining microvessel architecture at the network level and no opportunity for directly assessing microvessel function and RBC supply.

## Dynamics of Microvascular Network Reconstruction in Ischemic Muscle Revealed by Intravital Microscopy

One approach that offers both architectural and functional windows into the microcirculation is intravital video microscopy. This is because intravital microscopy affords spatial and temporal resolution suitable for tracking individual RBC transit while maintaining network-level integrity. Recently, we applied this real-time imaging strategy to delineate the angiogenesis process in the mouse skeletal muscle subjected to ischemic injury (Arpino et al., 2017). The aforementioned predilection of the distal hindlimb muscles for ischemic injury proved to be fortuitous, as these muscles, including the extensor digitorum longus (EDL), are easily accessible for live imaging.

**Figure 1:**
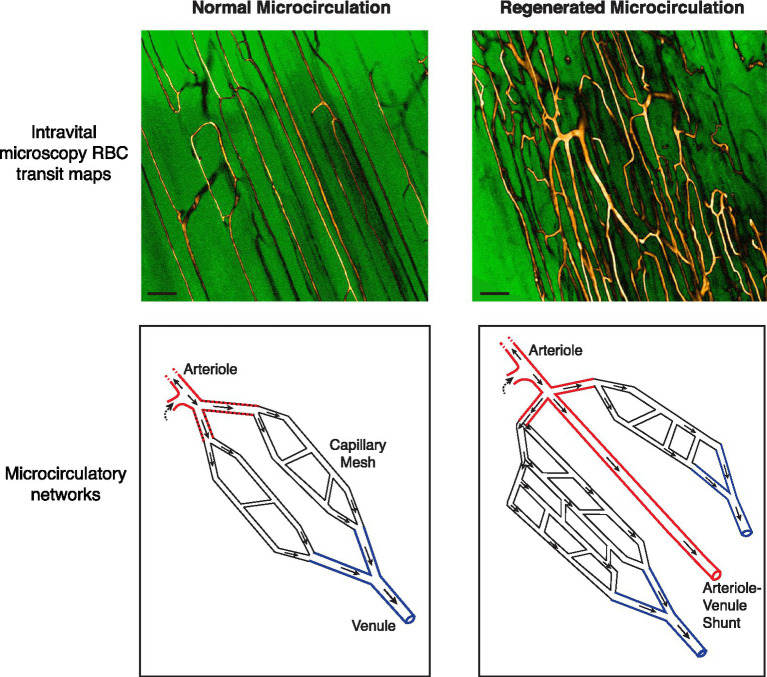
Abnormalities in the regenerated microvascular network in the mouse subjected to ischemic injury. (Top) Intravital microscopy-derived RBC transit maps of the extensor digitorum longus muscle in a C57BL/6 mouse. Maps depict all flow-receiving surface (50 μm deep) vessels over a 15 s period. Custom look-up tables were applied to grayscale image maps for enhanced contrast. A native network is shown in the left panel. Capillaries are running parallel to the skeletal myofibers with ordered anastomoses between parallel capillary segments. A regenerated network 28 days after ischemic injury is shown in the right panel, showing increased and chaotic vascularity. Bar, 100 μm (Bottom) Schematics of native and regenerated (day 28) microcirculatory networks. In the regenerated network the distal arteriole trifurcates. Moreover, one of the three limbs drains directly into a venule rather than branching into a capillary meshwork [See Arpino et al. (2017) for RBC transit maps and videos demonstrating these phenomena].

The assessment revealed several key insights about the speed and robustness with which the distal microcirculation can regenerate in muscle subjected to ischemic injury. The EDL muscle itself underwent widespread myofiber necrosis. 1 day after the injury, all or mostly all myofibers were pale staining, shrunken, and devoid of nuclei. This was followed in a matter of days by widespread myofiber regeneration. A similar necrosis-regeneration profile has been observed in the ischemic-injured soleus muscle (Paoni et al., 2002). Interestingly, the myofiber necrosis phase was associated with complete cessation of microvascular flow, at least to the depth that could be imaged microscopically (~50 μm). This flow cessation was not short-lived – it lasted for 4 days and was associated with immunohistochemical evidence for capillary obliteration. However, capillary destruction was followed by a rapid angiogenesis response. 10 days after injury, there was a dense neo-vascular network (Figure 1). This new network was hyper-vascular and relatively chaotic, with little evidence for arteriole – capillary – venule hierarchy (Arpino et al., 2017; Lee et al., 2020). Yet by day 14, there was remarkable remodeling, with branch pruning and realignment of capillaries along the muscle long axis. Moreover, the vessels had differentiated into terminal arteriole – capillary – post-capillary venule units. Interestingly, the distal-most arterioles were seen to course parallel to the muscle fibers, rather than orthogonal to them, running up to 1 mm before branching (Arpino et al., 2017). These findings revealed a remarkable capacity to regenerate the distal microvasculature, i.e., capillaries and terminal arterioles, in skeletal muscle subjected to severe ischemic muscle injury.

## Flaws in the Regenerated Microcirculation: Network Architecture Defects

Despite the capacity to rapidly regenerate a hierarchical distal microvascular network, the extent to which the network microanatomy is faithfully recreated also needs to be considered. Intriguingly, we have found that the fundamental principle of microvessels bifurcating as they branch was violated in the regenerated microcirculation (Arpino et al., 2017). Roughly 7% of regenerated terminal arterioles were found to either trifurcate or quadrificate, a pathologic feature observed in tumor vessels (Less et al., 1991; Fukumura et al., 2010; Figure 1). As well, among bifurcating neo-arterioles, the daughter branches could have unequal lumen diameters, not the symmetrical bifurcation of arterioles seen in normal muscle. Advances in deep, 3D vascular microimaging, such as with cleared tissue microscopy and microCT angiography (Frontini et al., 2011; Dunmore-Buyze et al., 2014; Zhang et al., 2018; Dunmore-Buyze et al., 2019; Becher et al., 2020) hold promise for further characterizing these peculiar abnormalities in PAD and animal models.

**Figure 2:**
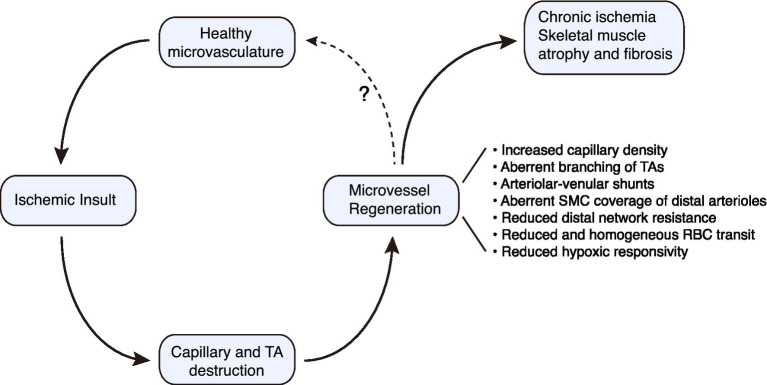
Conceptual paradigm for regenerative angiogenesis in the mouse hindlimb subjected to ischemic injury. Microvascular regeneration proceeds robustly following severe ischemic injury to skeletal muscle. However, there are structural and functional deficiencies that must be overcome if optimal tissue perfusion is to be restored. TA, terminal arteriole.

Also remarkable was that not all neo-arterioles diverged into a capillary meshwork. Instead, approximately one-third of all terminal arterioles directly connected to a venule, a form of arterial–venous (AV) malformation. However, unlike the widely studied congenital AV malformations (Park et al., 2009), these were micro-malformations – non-branched conduits of similar caliber to the terminal arteriole and scattered throughout the regenerated microvasculature. As well, because these relatively high-flow AV connections ran alongside capillaries, they can be expected to divert RBCs away from the nearby capillaries and compromise gas exchange (Ellis et al., 2002; Pries et al., 2010). Critically, none of these arteriolar abnormalities were transient and they were still evident 120 days after the ischemic insult (Arpino et al., 2017). Therefore, we propose that the regenerated microcirculation is vulnerable to a phenomenon of “arteriolar dysgenesis,” a set of abnormalities in the terminal arterioles that can be expected to compromise oxygen delivery (Figures 1, 2).

## Flaws in the Regenerated Microcirculation: Flow Control Defects

Blood flow to muscle following ischemic insult is commonly assessed using perfusion techniques, such as laser Doppler flow or laser speckle imaging (Limbourg et al., 2009; Frontini et al., 2011; Lee et al., 2020). This has established that hindlimb perfusion declines abruptly after femoral artery excision in the mouse and returns over the next 2–4 weeks, reaching 85~100% of that of the contralateral limb (Couffinhal et al., 1998; Scholz et al., 2002; Helisch et al., 2006). However, this type of assessment is a measure of bulk flow and does not have the spatial resolution to assess the wide range of RBC transit profiles in the microcirculation. Using intravital video microscopy, we have found that there are substantial, otherwise hidden, functional defects in the regenerated microcirculation. For example, RBC transit velocity in regenerated capillaries was well below that of the native microcirculation. 28 days after femoral artery excision, the RBC velocity in regenerated capillaries was only ~50% of that of normal capillaries. The low RBC transit velocities may partly reflect reduced peak pulse wave velocities in the upstream collateral vessels, but bulk flow typically returns close to baseline as assessed by laser Doppler techniques (Scholz et al., 2002; Helisch et al., 2006). Furthermore, at 120 days RBC velocity had actually declined to ~20% of normal, suggesting a widespread breakdown of RBC transit control (Arpino et al., 2017). Associated with this was a high prevalence of capillaries with the stalled flow. The sluggish or completely halted flow is particularly noteworthy, and given that the metabolic demands of regenerating skeletal myofibers would be expected to increase, not decrease (Koopman et al., 2014).

There was also a distinct loss in the normal heterogeneity of RBC velocities in the network, a fundamental feature of microvascular flow distribution in response to local metabolic demands (Duling and Damon, 1987; Ellis et al., 1994; Arpino et al., 2017). This monotony in RBC transit suggests that the regenerated microcirculation has lost its capacity to heterogeneously distribute RBCs in accordance with the spatial heterogeneity of the skeletal muscle requirements (Murrant et al., 2017; Fernando et al., 2019). Flow control malfunction was further suggested by studying the RBC delivery response to local hypoxia. Hypoxia is a powerful stimulus for enhancing the delivery of RBCs in skeletal muscle (Parthasarathi and Lipowsky, 1999; Ghonaim et al., 2011). However, this response was severely blunted in the regenerated microcirculation (Arpino et al., 2017). Together, the findings indicate that the post-ischemia regenerated network is compromised in its ability to tune the delivery RBCs to match local needs, in contrast to the well-orchestrated mechanisms for healthy networks, as summarized above.

## Flaws in the Regenerated Microcirculation: Defects in Contractile Machinery of the Distal Arterioles

There are several sites in the vasomotor control loop that could be vulnerable in regenerated muscle and potentially explain the abnormalities in flow control. While speculative, this includes defects in oxygen or metabolite sensing by the endothelium, impaired transmission of signal up the microvasculature, blunted communication between the endothelium and SMCs, reduced production or bioavailability of nitric oxide, and compromised sympathetic neural control (Ellis et al., 2005; Bagher and Segal, 2011; Clifford, 2011; Murrant et al., 2017). Another possibility that we have considered is the vasomotor competence of the terminal arteriole, a critical gatekeeper of RBC delivery.

Scanning electron microscopy has established that normal precapillary arterioles in the mouse are circumferentially wrapped by SMC processes (Holley and Fahim, 1983). A similar arrangement in brain arterioles has been delineated using genetically encoded mural cells (Hill et al., 2015). By reconstructing confocal optimal images, we found that distal arterioles in native mouse EDL muscle were blanketed by circumferential, SM α-actin-positive processes (Arpino et al., 2017). Three to five actin microfilament bundles could be seen within each process. In arterioles of 12–20 μm diameter, these processes were tightly packed with inter-process spaces of about 0.7 μm. Terminal arterioles 7–12 μm diameter were also circumferentially wrapped by SMC processes, although the processes were thinner and somewhat less aligned. In contrast, the SMC process wrapping of regenerated arterioles was distinctly disordered. There were bare zones up to 13 μm in length, and inter-process spacing was significantly increased overall. Haphazard mural cell coverage was still evident 56 and 120 days after injury (Arpino et al., 2017). These high-resolution 3D findings strongly suggest that disorder in SMCs and their processes around distal arterioles could underlie impaired vasomotor control in a regenerated microcirculation. It will also be important to ascertain the high-resolution cell structure and vasomotor function of upstream resistance vessels, from feed arteries to terminal arterioles. A time course of functional recovery of this part of the tree has recently been described following chemical injury (Fernando et al., 2019).

## Insights From Computational Modeling of a Regenerated Microvascular Network

Data from intravital microscopy can be further leveraged to address unanswered questions using computational modeling. One challenge in understanding microcirculatory dynamics is acquiring local hemodynamic parameters. Whereas critical hemodynamic attributes, such as resistance and shear stress can be readily derived from measurements made in large and medium-sized arteries (Guzzardi et al., 2015; Balint et al., 2019; Kwan et al., 2019), this cannot currently be done in microvessels. The small size of the vessels and intrinsic variability of the network morphology pose serious challenges. Computational modeling, using data from intravital microscopy and advanced microscopy, can help fill this gap.

Using intravital microscopy data, microvessel networks can be reconstructed into a collection of nodes and cylindrical vessel segments, and two-phase (RBC and plasma) steady-state blood flow simulations can be performed (Pries et al., 1990, 1994; Goldman and Popel, 2000; Arpino et al., 2017). Using such an approach, we found that the distal network resistance in the regenerated microvasculature was substantially lower than that in native muscle (Arpino et al., 2017).

Computational models can address other complexities of the microcirculation, including shedding light on the human microvasculature where the application of intravital microscopy is rare (Fisher et al., 2016). Models have evolved to be predictive and that can account for the non-linear rheology, changing hematocrits, viscosity, and the complex geometries of the microcirculation. Plasma skimming is one example of a biophysical process important to the microcirculation that has been tackled by advanced modeling (Lee et al., 2018; Possenti et al., 2019). This phenomenon entails phase separation at microvascular bifurcations. With the asymmetric bifurcation of an arteriole, the smaller daughter branch effectively “skims” the plasma-rich, cell-poor marginal layer of the blood. The result is different hematocrits and oxygen delivery in the two daughter branches (Popel et al., 1986; Pries et al., 1989, 1996). This aspect of flow heterogeneity is an intrinsic property of the microcirculation, not a random one (Pries et al., 1996). Therefore, understanding how phase separation at branches is impacted by ischemic muscle injury is of great interest. This is particularly so given the peculiar morphometry and branching of terminal arterioles, including trifurcations and quadrifications, in the regenerated microcirculation (Arpino et al., 2017). Understanding the impact of these anomalies could be advanced by computational model interrogation.

Multiscale microvascular models that encompass the vessel EC and SMC wall components also have great potential (Murfee et al., 2015; Zhang et al., 2016). Integrating data from these cell compartments with flow parameters and ischemia-induced growth factors could reveal novel insights into productive versus pathologic neovascularization. As well, because arterioles in regenerated microvessels can be partially denuded of SMCs, it will be important to determine how this influences flow reserve, i.e., the maximum increase in blood flow above resting flow, in the regenerated skeletal muscle. A network-wide assessment of SMC wrapping, lumen diameter, and vascular density throughout the arteriolar tree to inform computational models could provide key insights into the ability of a regenerated network to augment flow, for example, during exercise. A starting point for this could be cross-sectional arteriolar wall measurements. This, in turn, would require overcoming the sampling error inherent in histochemical assessments, given the number of arterioles and their size heterogeneity. In this regard, we have described a fully automated segmentation and vascular quantification algorithm for whole-slide images immunostained for smooth muscle α-actin, as well as a machine learning-based strategy to classify and differentiate arterioles from venules (Elkerton et al., 2017; Xu et al., 2017). 3D histology reconstruction methods could further inform computational models of blood flow (Xu et al., 2015).

## Translational Considerations

Several considerations must be kept in mind when framing advances described herein to the scenario of patients with PAD. First, mouse models of acute arterial occlusion should not be viewed as models of PAD per se, but rather as models of critical processes that proceed in patients with PAD. In this perspective, we have focused on microvascular regeneration following ischemic insult. Acute arterial occlusion in mice provides a rich opportunity to interrogate this regenerative response. Moreover, vessel occlusion is indeed a feature of PAD, although generally not as a singular event. As well, occlusive events can occur throughout the vascular tree and on a background of chronic atherosclerotic narrowing (Narula et al., 2018). Another context for the findings discussed in this review is therapeutic angiogenesis for PAD. The mouse data tell us that even with robust angiogenesis, skeletal myofibers may not receive their oxygen and nutrient needs because the structural and functional competence of the regenerated microvessels is impaired. Thus, we propose that microvascular normalization as a therapeutic strategy merits attention. As a proof-of-concept, enhancing SMC coverage of distal arterioles, one of the vulnerabilities in the regenerated microvasculature, has been undertaken using a local growth factor delivery strategy (Said et al., 2019).

## Summary

Regenerated microvessels in skeletal muscle are a reality for many individuals with advanced PAD and a potential therapeutic opportunity (Hammarsten et al., 1980; Ho et al., 2006b; Chevalier et al., 2020). Although classically a blind spot in vascular disease, an understanding of the structure and function of a regenerated microvascular network in muscle is emerging. There is a remarkable capacity for microvessel network regeneration after ischemic injury, at least for distal arterioles and capillaries. However, when this network regenerates there are a number of important, almost hidden, flaws in architecture and flow control. The consequences include a neo-circulation with impaired perfusion control and blunted responsivity (Figure 2). These microcirculatory pathologies could contribute to the progressive nature of PAD and its refractoriness to therapy. Building on the findings and strategies described herein to probe the regenerated microcirculation, and the molecular underpinnings of its defects will be critical for normalizing the regenerated microvasculature in at-risk patients.

## Ethics Statement

The animal study was reviewed and approved by The University of Western Ontario, Animal Care Committee, University Council on Animal Care.

## Author Contributions

HY, J-MA, and JL drafted the manuscript. JGP revised the manuscript and supervised the work. All authors contributed to the article and approved the submitted version.

### Conflict of Interest

The authors declare that the research was conducted in the absence of any commercial or financial relationships that could be construed as a potential conflict of interest.
